# Integrated Microfluidic Giant Magnetoresistance (GMR) Biosensor Platform for Magnetoresistive Immunoassay of Myoglobin

**DOI:** 10.3390/bios16010008

**Published:** 2025-12-22

**Authors:** Yikai Wang, Huaiyu Wang, Yunyun Zhang, Shuhui Cui, Fei Hu, Bo’an Li

**Affiliations:** 1 Science and Technology Research Center of China Customs, Beijing 100026, China; 2 State Key Laboratory for Manufacturing System Engineering, School of Instrument Science and Technology, Xi’an Jiaotong University, Xi’an 710049, China; 3 Department of Clinical Laboratory, The Fifth Medical Center, Chinese People’s Liberation Army (PLA) General Hospital, Beijing 100039, China

**Keywords:** acute myocardial infarction, myoglobin, giant magnetoresistance (GMR) sensor, microfluidic technology, magneto-immunoassay

## Abstract

Acute myocardial infarction (AMI) is a rapidly progressing cardiovascular condition associated with high mortality. Myoglobin is an early biomarker of AMI; however, its detection using conventional methods is limited by complex workflows and low resistance to interference. In this study, we developed an integrated myoglobin detection platform that combined magneto-immunoassay with microfluidic technology. A giant magnetoresistance (GMR) sensor was fabricated using micro-electro-mechanical systems (MEMS). The designed microfluidic chip integrated sample pretreatment, immunoreaction, and magnetic signal capture functionalities. Its built-in GMR sensor, labeled with magnetic nanoparticles, directly converted the “antigen–antibody” biochemical signal into detectable magnetoresistance changes, thereby enabling the indirect detection of myoglobin. A magneto-immunoassay analysis system consisted of a fluidic drive, magnetic field control, and data acquisition functions. Various key parameters were optimized, including EDC/NHS concentration, antibody concentration, and magnetic bead size. Under the optimal conditions and using 1 μm magnetic beads as labels and an external detection magnetic field of 60 Oe, the platform successfully detected myoglobin at 75 ng/mL with ∆MR ≥ 0.202%. Specificity tests demonstrated that the platform had high specificity for myoglobin and could effectively distinguish myoglobin from bovine serum albumin (BSA) and troponin I. This study presents a rapid, accurate myoglobin detection platform that can be applied for the early diagnosis of AMI.

## 1. Introduction

Acute myocardial infarction (AMI) is a critical condition caused by acute coronary artery occlusion that leads to reduced myocardial blood supply and subsequent necrosis. The clinical symptoms of AMI include persistent retrosternal pressure-like pain or chest tightness lasting more than 30 min, and these symptoms may rapidly progress to severe complications such as heart failure, arrhythmias, and cardiogenic shock [1,2,3]. Without prompt intervention, the risk of sudden cardiac death remains high. Diagnosis generally involves coronary angiography, electrocardiography (ECG), cardiac ultrasound, and cardiac injury markers. Among all, cardiac injury markers are among the most effective approaches for the early detection and monitoring of AMI [4]. Myoglobin is a small oxygen-binding protein with a molecular weight of 17.5 kDa. Serum myoglobin is one of the earliest markers to appear following acute myocardial infarction, with levels commencing to rise within 1–3 h post-onset, peaking at 6–7 h, and returning to baseline within one day [5]. Myoglobin possesses a 100% negative predictive value: within 2 to 12 h of chest pain onset, a negative test result excludes AMI. Peak elevation correlates directly with the extent of myocardial injury or necrosis and its prognosis; a higher peak indicates greater damage or necrosis. Due to its long half-life in blood, myoglobin is a suitable indicator for early AMI diagnosis [6,7]. Conventional myoglobin detection methods include immunoturbidimetric assays, fluorescent immunoassays, chemiluminescent immunoassays, latex agglutination tests, and enzyme-linked immunosorbent assays (ELISA). However, the methods above all exhibit significant limitations: not only do they require substantial costs and advanced equipment, but they also rely on professionally trained operators. Furthermore, these methods remain deficient in terms of sensitivity and specificity, whilst also presenting issues such as time-consuming detection procedures and the necessity for sample pre-treatment [8,9].

In recent years, the detection efficiency and sensitivity of magnetoresistive sensors have been continuously improved. Their cost, power consumption, and physical dimensions have been decreased, while their advantages, such as ease of integration and portability, have been preserved [10,11,12]. For these reasons, magnetoresistive sensors have attracted growing research interest. In 1996, Kriz [13] reported the first magnetic sensors for biological detection. Wang [14] subsequently reported a sensor with quantitative analysis capacity in which magnetic bead-labelled biomolecules were introduced into coils, and the output was measured based on changes in inductance. Advancements in MEMS technology have enabled the miniaturization via micro-nano fabrication techniques of various magnetic sensors, including anisotropic magnetoresistance (AMR), GMR, tunnel magnetoresistance (TMR), and miniature fluxgate sensors. These sensors have been widely applied in biodetection [15]. In 2013, Issadore D et al. [16] reported a Hall sensor labelled with MnFe_2_O_4_ magnetic beads for detecting bacteria such as *Staphylococcus aureus* and *Micrococcus luteus*, achieving the detection of individual bacteria. In 2011, Teresa et al. [17] integrated dextran-coated Fe_3_O_4_ magnetic nanoparticles (with sizes of 50, 130, and 250 nm) with a SQUID sensor and a loop-amplification system. The sensor has a low detection limit of 4 pmol/L and can detect DNA within 20 min. In 2014, Wang et al. developed a zigzag, sandwich-type thin-film giant magnetoimpedance (GMI) biosensor that, when coupled with magnetic bead labelling, can quantitatively detect AFP and CEA antigens with sensitivities of 1 pg/mL and 10 ng/mL, respectively [18]. GMR biosensors have many advantages, including simple fabrication processes, stable performance, excellent mechanical properties, wide temperature range, and high detection specificity [19]. Kenaan et al. (2020) achieved direct simultaneous detection of troponin, procalcitonin, and C-reactive protein in undiluted urine using GMR sensors, with detection limits reaching 10 pg/mL [20]. Sun et al. (2025) developed a highly sensitive magnetic biosensor for bacterial detection by exploiting magnetic field line convergence effects, achieving a detection limit of 10 CFU/mL [10]. They have been extensively applied to detect nucleic acids [10,21,22], viruses [23], tumor markers [24], and myocardial injury markers [25,26]. Nevertheless, most current GMR-based assays remain heavily dependent on manual operation and lack dedicated integrated instrumentation; thus, their potential for automation and broader system integration is limited.

Microfluidic technology, first proposed by Manz et al. in 1990 [27], has since driven significant advances in biomedical research and analytical chemistry due to its capacity for integration and miniaturization. In 2006, Aytur et al. [28] integrated GMR sensors into a microfluidic chip to measure magnetic fluid flow rates and count magnetic droplets. Later, in 2015, Kokkinis et al. [29] designed a microfluidic chip that can be actuated by a gradient magnetic field for manipulating magnetic-labelled particles. By combining GMR sensor signals with Fourier transform analysis, they were able to qualitatively detect *Escherichia coli*. Yang et al. [24] further expanded the field by developing a GMR-based magneto-immunoassay platform with multi-target detection capacity. The platform can simultaneously detect 12 tumor markers. However, this platform has a complex structure and low stability, and with its single-use chips, it has low versatility and reproducibility. Feng Z. et al. [30] (2019) reported an integrated GMI biosensor-based microfluidic chip that can detect prostate-specific antigen (PSA) within 40 min. The chip has a high sensitivity of 0.1 ng/mL and a wide dynamic range of 0.1 ng/mL to 20 ng/mL. To achieve simultaneous plasma separation and label-free albumin detection, Li et al. (2024) developed a microfluidic chip platform integrating a GMR sensor, demonstrating a detection limit of 0.16 μg/mL for recombinant albumin [31]. Despite its high potential for automation, this chip has many limitations, including lengthy detection cycles and low system stability.

**Figure 1:**
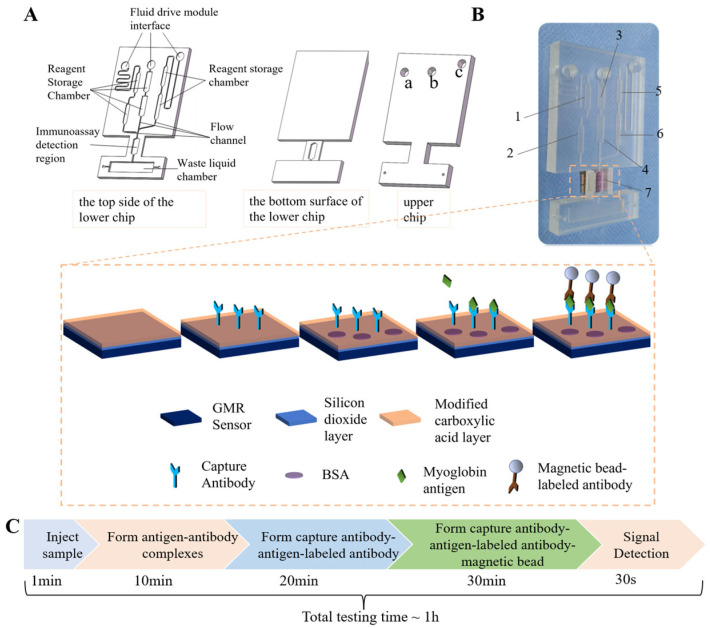
Schematic diagrams showing the detection principle of the magnetic immunoassay on a microfluidic chip. (**A**) Structure of the microfluidic chip. (**B**) Photograph of the actual microfluidic chip. The magnetic immunoassay takes place in the detection zone highlighted by a dotted box. The GMR sensor surface is passivated with a SiO_2_ layer. Carboxyl groups on the surface are activated using EDC/NHS in PBS to enable antibody immobilization, and the remaining sites are blocked with BSA to minimize non-specific binding. As a sample containing myoglobin flows through the detection zone, the target antigen is captured by the immobilized antibodies. Detection antibodies pre-conjugated to magnetic beads then bind to the captured antigen, anchoring the beads to the sensor surface. Accumulation of beads perturbs the local magnetic field at the GMR sensor surface, resulting in a measurable change in magnetoresistance that reflects the presence and concentration of the target antigen. (**C**) Detection procedure and time.

To address the limitations of the current magneto-immunoassay systems, we integrated magneto-immunoassay with microfluidic technology and developed an accurate, user-friendly myoglobin detection platform (Figure 1). To overcome the fragmented functionality and cumbersome operation of conventional methods, a microfluidic chip integrating sample pretreatment, immune reaction, and magnetic signal capture was developed (Figure 1A,B). To further streamline the operational workflow, the chip was coupled with a GMR sensor to allow the simultaneous reaction and detection. To enhance the precision and reduce detection errors, various parameters affecting the chip performance were optimized. Overall, this work presents an integrated approach that addresses challenges in magneto-immunoassays, offering a comprehensive solution for the rapid, accurate, and convenient detection of myoglobin.

## 2. Materials and Methods

### 2.1. Reagents

Human myoglobin antigen, mouse anti-myoglobin monoclonal antibodies, and fluorescein isothiocyanate (FITC)-labelled mouse anti-myoglobin monoclonal antibody were obtained from Shanghai Lingchao Bio-New Materials Co., Ltd (Shanghai, China). Bovine serum albumin (BSA) was purchased from Wuhan Boshide Bio-Technology Co., Ltd (Wuhan, China). Poly(allylamine hydrochloride) was obtained from Shanghai McLean Biochemical Technology Co., Ltd (Shanghai, China). Maleic anhydride was purchased from Sigma-Aldrich (St. Louis, MO, United States). 1-(3-Dimethylaminopropyl)-3-ethylcarbodiimide hydrochloride (EDC) and N-hydroxysuccinimide (NHS) were purchased from Aladdin Reagent Co., Ltd (Shanghai, China). Phosphate-buffered saline (PBS) was acquired from Shanghai Biyuntian Biotechnology Co., Ltd (Shanghai, China). Superparamagnetic beads (diameters = 300 nm, 1 μm, and 2.8 μm) were obtained from Shanghai Lingchao Bio-New Materials Co., Ltd (Shanghai, China).

### 2.2. Fabrication of Sensor

The GMR sensor was fabricated using standard MEMS methods. Each sensor chip was incorporated with two distinct detection units (each with dimensions of 2 mm × 3 mm): a magnetoresistive signal detection unit (primary unit) and a reference unit. Each detection unit comprises seven GMR resistive strips, each with a width of 200 μm. Electrodes (1.5 mm × 4.5 mm) were attached to both ends of the resistive strips. The sensitive axis of the sensor was positioned perpendicularly to the line connecting these electrodes. A 50-nm-thick SiO_2_ layer, which serves a dual purpose: separating the sensing elements from the liquid reagents and providing a substrate for the subsequent immobilization of capture antibodies, was sputtered on the active sensor surface. A Helmholtz coil was used to make the magnetic field strength applied to the GMR biosensor tunable. The magnetic field strength was controlled by changing the current supplied to the coil. The sensor was positioned at the center of the Helmholtz coil. A gaussmeter was used to calibrate the magnetic field strength and ensure that the sensitive axis of the sensor aligned with the direction of the applied magnetic field.

### 2.3. Fabrication of Microfluidic Chip

The H-shaped microfluidic chip (Figure 1A,B) had overall dimensions of 46 mm × 75 mm and was composed of two layers: an upper and a lower layer with a thickness of 6 mm and 2.5 mm, respectively. All microchannels and functional chambers were fabricated within the lower layer. The chip was incorporated with several key functional components, including reagent reservoirs, microchannels, an immunocapture detection zone, a waste reservoir, vent holes, and interfaces for external fluidic drive modules. Three fluidic drive interfaces (a, b, c in Figure 1A) were placed on the upper layer as circular ports, each with a diameter of 5 mm. The leftmost port (a) served as the sample inlet connecting with the external fluidic drive system. The microchannels consisted of a square cross-section with dimensions of 500 μm × 500 μm. Two reagent reservoirs (Zones 3 and 4) in the central region of the chip were used to store washing buffer for cleaning the immunocapture detection zone. In the lower left region, a hexagonal chamber was used to store the lyophilized labelled antibody (Zone 2). The solvent reservoir (Zone 1), adjacent to Zone 2, was used to store phosphate-buffered saline (PBS) for sample dilution and reconstitution of lyophilized antibodies. The magnetic bead storage chamber (Zone 6; lower right corner) was constructed to store lyophilized magnetic beads. The solvent reservoir (Zone 5) was used to store PBS for the reconstitution of magnetic beads. The specific immune reaction and subsequent magnetic signal detection occurred in Zone 7, which is defined as the immunocapture detection zone. This is achieved by integrating a GMR sensor directly on the reverse side of the microfluidic chip in this region. Vent holes were connected to the waste reservoir to balance flow resistance across the chip.

### 2.4. Construction of the Experimental Platform

The magneto-immunoassay platform was constructed as an integrated system composed of several coordinated functional modules, including the magnetoresistive sensor, microfluidic chip, fluid drive module, magnetic field control module, temperature control module, data acquisition module, and host computer (Figure S1). The magnetoresistive sensor served as the primary detection element and operated by transducing changes in the local magnetic bead concentration on the sensor surface before and after the immunoassay into corresponding changes in the electrical resistance. These resistance changes served as an indirect quantitative indicator of the immunoassay reaction. The microfluidic chip provided the structured microenvironment necessary for automated key assay steps, including sample injection, reagent mixing, immunoreaction, and washing, within a confined fluidic architecture to minimize cross-contamination and improve overall detection throughput.

Flow manipulation within the microchannels was achieved using the fluid drive module to precisely regulate flow rate, velocity, and timing to ensure uniform reagent mixing, controlled reaction progression, and efficient washing of the immunocapture zone. The magnetic field control module was attached to apply a stable and tunable external magnetic field essential for consistent magnetic actuation of beads and maintaining the sensitivity of the detection. Temperature stability was maintained by the temperature control module, which held the platform at 37 °C to preserve reaction kinetics and prevent temperature-induced variations. The data acquisition module was utilized to simultaneously monitor the background magnetic field and the magnetoresistive signal, capture stray magnetic field perturbations produced by bead movement, and translate them into measurable resistance fluctuations as analogue signals. Analogue signals were subsequently amplified, filtered, digitized, and transmitted to the host computer for analysis and interpretation.

### 2.5. Simulation of Microfluidic Chip

A pneumatic drive system was employed to control the fluid flow in the microfluidic chip. Various fluid drive parameters were calculated to determine the effects of gas compressibility, caused by significant gas volume changes as a result of elevated internal pressures, on system performance. Gas volume changes were considered negligible at very low operating pressures, and the gas was approximated as an incompressible fluid. In the evaluation of the drive characteristics, the pressure distribution within the microfluidic chip was simulated. The equivalent diameter and Reynolds number of the microchannels were calculated to ensure laminar flow. The simulations were performed using COMSOL Multiphysics^®^ 6.0 software, and the pressure field and velocity distribution within the chip were determined.

### 2.6. Workflow for Microfluidic Chip

The myoglobin detection was performed following a sequential protocol using the integrated GMR-microfluidic platform. First, 20 μL of the sample to be tested was injected into port (a), and the sample flowed through Reagent Reservoir 1 where it was diluted with pre-stored PBS buffer, a process that took 1 min (Step i). Subsequently, the diluted sample passed through Reagent Reservoir 2 and mixed with the reconstituted lyophilized labeled detection antibody, resulting in the formation of myoglobin–antibody complexes, with this incubation step lasting 10 min (Step ii). The resulting mixture was then transported to the immunocapture detection zone, where myoglobin antigens were specifically captured by the pre-immobilized capture antibodies on the surface of the GMR sensor, thereby forming “capture antibody-antigen-labeled detection antibody” sandwich complexes; this immunocapture process required 20 min (Step iii). After that, washing buffer from Reagent Reservoir 3 was introduced via port (b) to eliminate unbound labeled detection antibodies, which was completed within 30 s (Step iv). Next, the solvent in Reagent Reservoir 5 was injected through port (c) to reconstitute the lyophilized magnetic beads in Reagent Reservoir 6 and deliver the reconstituted magnetic beads to the detection zone; the magnetic beads bound to the Fc region of the labeled detection antibody, thus completing the formation of “capture antibody-antigen-labeled detection antibody-magnetic bead” complexes, and this step took approximately 30 min (Step v). Finally, a secondary washing was conducted using the buffer from Reagent Reservoir 4 to remove unbound magnetic beads, and the preparation of the detection complexes on the GMR sensor was finalized. The GMR biosensor then detected the magnetic signal perturbation induced by the captured magnetic beads to achieve qualitative analysis of the myoglobin antigen concentration in the sample, with the combined duration of the secondary washing and signal detection steps being 1 min (Step vi).

### 2.7. Magnetic Immunoassay Experiment

The GMR biosensor surface was first pretreated by sequential ultrasonic cleaning in acetone, anhydrous ethanol, and isopropanol. The sensor was then dried in an oven at 45 °C and subjected to UV irradiation for sterilization. To functionalize its surface, the sensor was treated with 50 μL of 1% (*w*/*v*) poly (allylamine hydrochloride) (PAH) for 5 min, rinsed with deionized water, and then incubated at 120 °C. To form a carboxyl-functionalized layer on its surface, the sensor was incubated in 2% (*w*/*v*) maleic anhydride aqueous solution for 5–10 min. To activate the carboxyl groups on its surface, the sensor surface was treated with 50 μL of PBS buffer containing 5% EDC (1-(3-dimethylaminopropyl)-3-ethylcarbodiimide hydrochloride) and 5% NHS (N-hydroxysuccinimide), and then washed with PBS and deionized water. Subsequently, 0.7 mg/mL capture antibody was applied on the sensor surface and incubated at 37 °C for 40 min to allow immobilization. To minimize non-specific reactions, the sensor surface was treated with a blocking agent (50 μL of 1% BSA in PBS) for 40–60 min. For magnetic labelling of the antibody, the labelled antibody was incubated with biotinylated magnetic beads for 60 min, during which the mixture was shaken every 10 min.

In magnetic immunoassay detection, PBS solutions containing myoglobin antigen at different concentrations were incubated with the above mixture at 37 °C for 20 min and then washed, followed by magnetically labelled antibody at 37 °C for another 30 min. Quantification of the target antigen was achieved through the GMR biosensor, which detected changes in magnetic signals.

Due to the inherent batch-to-batch variations in the baseline resistance of the fabricated GMR sensors, the sensor response was normalized using the magnetoresistance (MR) ratio, which can be calculated by Equation (1).

(1) $$MR=\frac{r_{i}-r_{0}}{r_{0}}\times 100\%$$

where MR is the magnetic resistance change rate, $r_{0}$ is the resistance value of the GMR sensor in the absence of an external magnetic field (Ω), and $r_{i}$ is the resistance value of the GMR sensor in the presence of an external magnetic field (Ω).

The sensitivity of the GMR magnetic sensor to changes in magnetic beads was determined based on the relative magnetic resistance change rate (ΔMR), which can be calculated using Equation (2).

(2) $$∆{MR}_{J}={MR}_{0}-{MR}_{J}$$

where ${MR}_{J}$ is the magnetic resistance change rate of the GMR sensor at a bead concentration of J ng/mL under an external magnetic field of 60 Oe, and ${MR}_{0}$ equals 1.8%.

### 2.8. Optimization of Fluorescence Immunoassay Conditions

The fluorescent immunoassay was carried out as follows. First, capture antibodies were immobilized on the SiO_2_ detection surface via spin coating and then blocked with BSA (to minimize non-specific binding). Subsequently, the sample was introduced to the immobilized antibodies. A solution containing fluorescently labelled antibodies was then flowed through the chamber. Finally, the fluorescent signal in the detection zone corresponding to the amount of the target antigen was measured using fluorescence microscopy. Key parameters influencing the performance of the developed immunoassay were systematically optimized. These parameters included the EDC/NHS mass ratio (2.5%, 5%, 7.5% and 10%), the capture antibody concentration (0.1 mg/mL, 0.4 mg/mL, 0.7 mg/mL, and 1 mg/mL), and magnetic bead size (300 nm, 1 μm, and 2.8 μm).

## 3. Results and Discussion

### 3.1. GMR Biosensor

The GMR biosensor platform detects specific biomolecular interactions by transducing the binding of superparamagnetic nanoparticles into measurable resistive signals. Capture antibodies are first immobilized on the active region of the GMR sensor through surface functionalization (Figure 1). The sensor surface is sputter-coated with a 50 nm SiO_2_ layer, which serves as a protective barrier and provides a substrate for antibody immobilization. When the sample flows through the detection zone, the target antigen is captured by the surface-bound antibodies. Magnetic beads conjugated with detection antibodies are then introduced, and the binding between these beads and the captured antigen forms a stable ‘capture antibody–antigen–detection antibody–magnetic bead’ sandwich complex that anchors the beads to the sensor surface.

The accumulation of superparamagnetic beads causes a localized magnetic field perturbation and, in turn, induces a measurable change in the electrical resistance of the GMR sensor. These magnetoresistance changes reflect the presence and concentration of the target antigen. This platform could be further developed and modified for the simultaneous detection of multiple target antigens.

**Figure 2:**
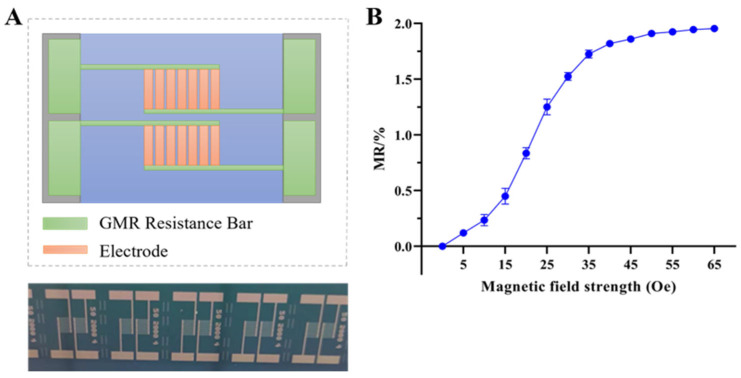
Fabrication and performance of the GMR sensor. (**A**) Schematic diagram (top) and photograph (bottom) of the GMR sensor. (**B**) Performance of the GMR sensor illustrated as a plot of the MR ratio as a function of magnetic field strength. Data represent the mean ± standard deviation (SD) from three independent measurements (n = 3).

As shown in Figure 2A, the GMR sensor was fabricated using standard MEMS processes. Each sensing unit contains seven GMR strips, each with a width of 200 μm. Electrodes (1.5 mm × 4.5 mm) are attached to both ends of the strips, with the sensitive axis of the strips oriented perpendicular to the line. Sensor performance was characterized based on the resistance measured using a digital multimeter as the strength of the external magnetic field was progressively increased from 0 to 65 Oe. As shown in the magnetic field-resistance (He-MR) curve (Figure 2B), the baseline resistance was approximately 950 Ω, and the maximum magnetoresistance ratio reached 1.9%. These results demonstrate that the GMR sensor was highly sensitive to changes in the magnetic field.

### 3.2. Simulation of Microfluidic Chip and Performance Test

**Figure 3:**
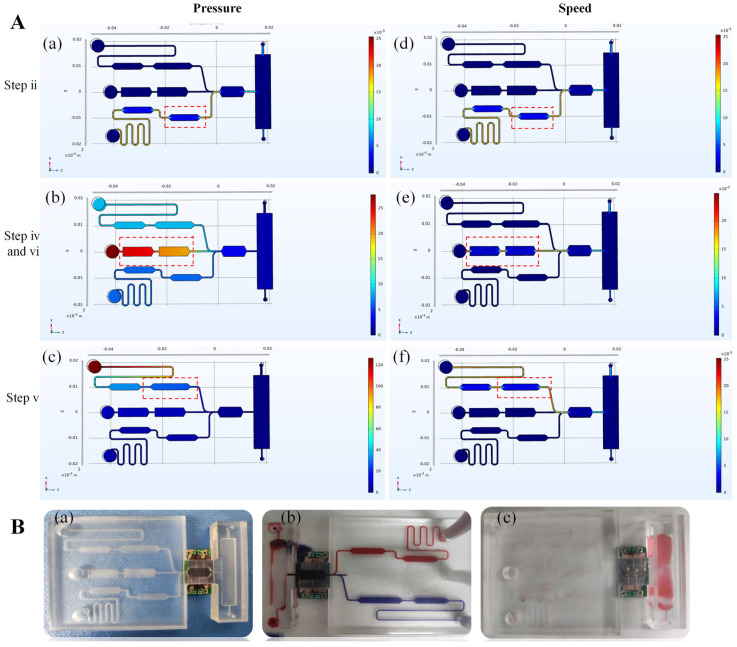
Microfluidic chip design, simulation, and functional validation. (**A**) CFD simulation results: (**a**–**c**) simulated pressure distribution across the chip, and (**d**–**f**) simulated velocity distribution across the chip. (**B**) Experimental validation of chip functionality: (**a**) photograph showing the assembled chip and airtight interface with the GMR sensor. (**b**) Visualization of fluid mixing in the detection zone, demonstrated using red and blue inks. (**c**) Demonstration of washing efficiency, illustrated by the complete removal of residual ink from the detection chamber.

A pneumatic drive system was used as a fluid control for the microfluidic chip. During operation, the pneumatic system interfaces with the chip’s fluidic network to regulate the movement of reagents. Because internal pressure distribution and flow velocity can directly influence the assay performance, computational fluid dynamics (CFD) simulations were performed to evaluate these parameters. Figure 3A shows the simulated pressure field and velocity distribution, respectively. In Step ii (Figure 3A(a,d)), the antigen flows into the antibody reservoir. In Steps iv and vi (Figure 3A(b,e)), which are the washing steps, the wash buffer is introduced. In Step v (Figure 3A(c,f)), the antibody-conjugated magnetic beads are introduced into the immunocapture detection zone. The simulation results indicate that each functional chamber operated hydraulically and independently, with minimal cross-talk. Flow resistance was mainly confined to the microchannels. The maximum simulated internal pressure was below 140 Pa, and the maximum flow velocity was approximately 2 × 10^−2^ m/s. These low-pressure conditions validate the suitability of treating the driving gas as an incompressible fluid. The simulations also confirmed that laminar flow was maintained throughout the operation, ensuring stable and predictable fluid behavior.

The chip was fabricated from PMMA using CNC milling, and the upper and lower layers were attached using adhesive. The GMR sensor was embedded in a recessed groove in the reaction zone to facilitate the assembly of the GMR sensor and microfluidic chip and ensure complete contact between the sensing unit and the reaction chamber during the assay, as well as to keep the sensor electrodes exposed for convenient signal detection. Functional testing confirmed excellent airtightness, with no leakage observed under operating pressures (Figure 3B(a)). Upon introduction into the detection zone, red and blue inks were mixed uniformly to form a homogeneous deep purple color (Figure 3B(b)). The chamber was completely cleared of ink residues after a standard washing step, which is demonstrative of efficient fluid exchange (Figure 3B(c)). These observations indicate that the chip has reliable fluidic control, mixing, and washing, and is properly integrated with the sensor.

### 3.3. Optimization of Experimental Conditions

**Figure 4:**
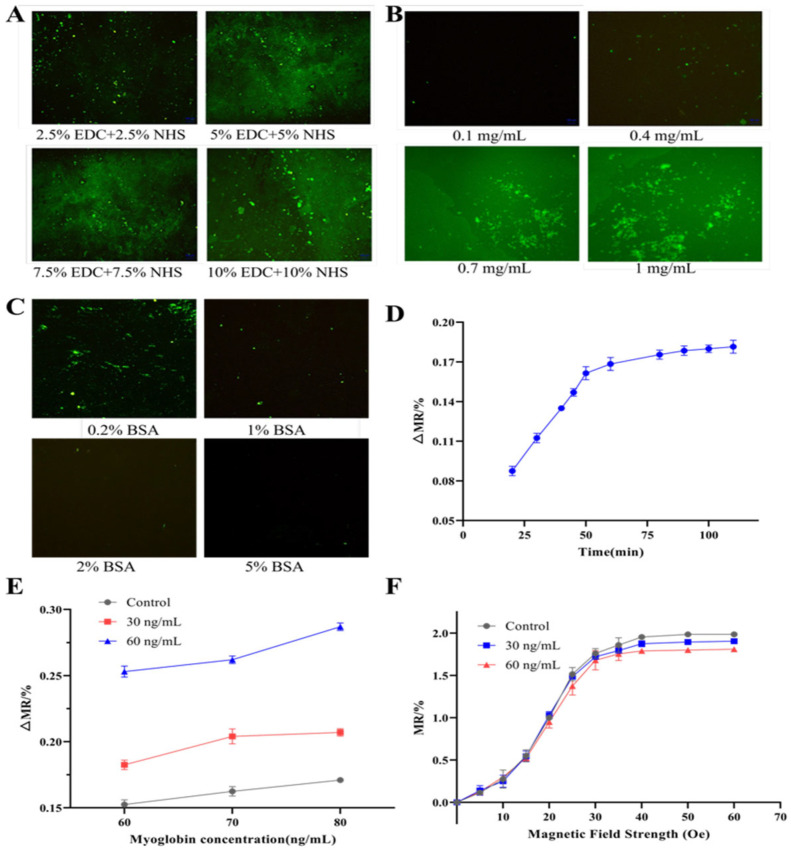
Optimization of key experimental parameters for the GMR-based magneto-immunoassay: (**A**) EDC/NHS mass fraction; (**B**) capture antibody concentration; (**C**) BSA concentration; (**D**) assay reaction time; and (**E**) magnetic bead size. The assay was performed using myoglobin antigen with concentrations of 60, 70, and 80 ng/mL (3 × 3 matrix). (**F**) Optimization of external magnetic field strength (He). PBS was used as the blank control. Superparamagnetic bead suspensions at 30 and 60 μg/mL were used as positive controls.

A mixture of EDC and NHS in PBS was used to activate surface carboxyl groups for covalent immobilization of antibodies. As the mass fractions of EDC and NHS directly affect the activation efficiency and, in turn, the density of the immobilized capture antibodies, we first optimized this mass fraction. As shown in Figure 4A, the fluorescence immunoassay demonstrated that the 2.5% EDC + 2.5% NHS condition produced significantly lower fluorescence intensity than the other three groups. In contrast, similar fluorescence intensities were obtained under the 5% EDC + 5% NHS, 7.5% EDC + 7.5% NHS, and 10% EDC + 10% NHS conditions. As the mass fraction of EDC and NHS increased, the fluorescence signal did not further increase, indicating that the 5% EDC + 5% NHS condition is sufficient for complete activation. Higher concentrations resulted in minimal improvement. Therefore, 5% EDC + 5% NHS was considered the optimal fraction and selected for all subsequent experiments. The concentration of the capture antibody determines the density of available myoglobin antigen binding sites in the detection zone. Insufficient antibody concentration can limit antigen binding capacity and reduce detection sensitivity, whereas excessive concentration can increase cost and may promote non-specific binding. As shown in Figure 4B, signal intensities for the 0.1 mg/mL and 0.4 mg/mL groups were significantly lower than those for the 0.7 mg/mL and 1.0 mg/mL groups. This indicates that lower concentrations lead to incomplete surface coverage. The 0.7 mg/mL and 1.0 mg/mL groups were not significantly different, indicating that the binding sites were saturated at 0.7 mg/mL. Considering detection sensitivity and cost, and to ensure complete binding saturation, 1.0 mg/mL was selected as the optimal capture antibody concentration. Further optimization identified 1% BSA as the optimal blocking concentration (Figure 4C). The immunoassay time was also optimized, and the data showed that the signal reached saturation after approximately 50 min and did not significantly increase with longer incubation (Figure 4D). Thus, a 50 min reaction time was considered optimal and adopted in subsequent experiments.

To evaluate detection performance near the clinical cut-off for human serum myoglobin (75 ng/mL), we tested myoglobin standards at 60, 70, and 80 ng/mL using magnetic beads with diameters of 300 nm, 1 μm, and 2.8 μm. As shown in Figure 4E, a clear positive correlation between ΔMR (relative change of magnetoresistance) and myoglobin concentration was observed for all three bead sizes. The 1 μm beads showed significantly higher detection sensitivity and stronger signal response compared to the 300 nm beads. Although the 2.8 μm beads generated a signal with the highest ΔMR, they had a high aggregation tendency, particularly at higher analyte concentrations, and thereby could increase the risk of signal overestimation and false-positive results. Thus, 1 μm magnetic beads were selected as the optimal beads.

The GMR sensor showed a low MR response when the external magnetic field at 0–10 Oe was applied. At the 10–30 Oe range, the response was discernible, but the signal-to-noise ratio remained insufficient to differentiate samples from the blank control (Figure 4F). A clear and statistically significant difference between the sample and the blank signals was observed in the 30–65 Oe range, despite a moderate decrease in absolute sensitivity. This range was thus considered the optimal range for the external magnetic field. Considering the balance between signal resolution and stable magnetic field generation, an external magnetic field strength of 60 Oe was selected for all subsequent detection experiments.

### 3.4. Detection Performance of the Magnetic Immunoassay Platform

**Figure 5:**
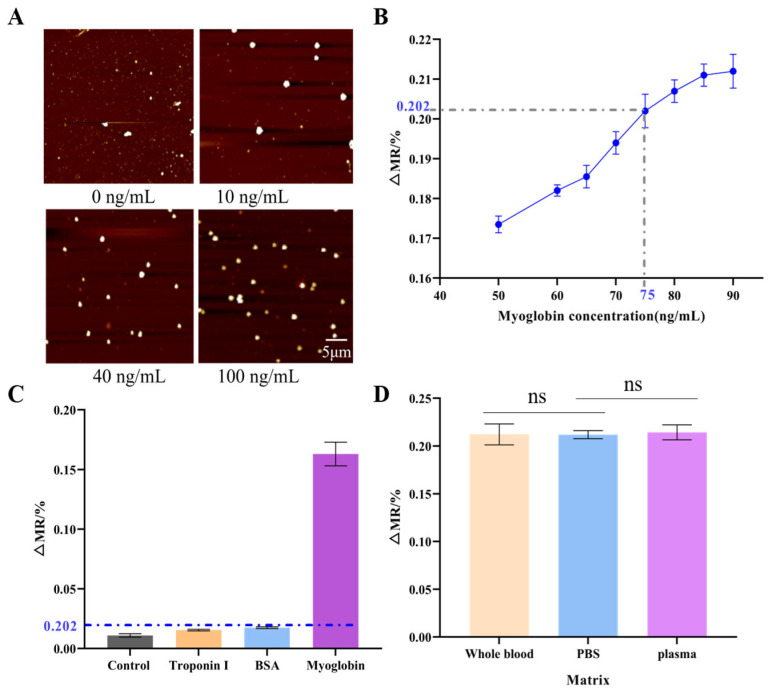
Detection performance of the magneto-immunoassay platform. (**A**) Atomic force microscopy (AFM) image showing the concentration-dependent accumulation of magnetic beads on the sensor surface. (**B**) Dose–response curve of the measured magnetoresistance (ΔMR) as a function of myoglobin concentration. (**C**) Specificity detection diagram. (**D**) The Effect of Complex Matrices on Reactions. ns indicates *p* > 0.05.

The integrated magneto-immunoassay platform developed in this work consists of various key elements: a core detection module, a fluidic drive system, a temperature control unit, a signal acquisition circuit, a central controller, a host computer, and an external magnetic field generator (Figure S1). To validate the capture performance of the immunocapture detection zone for magnetic beads, myoglobin antigen samples at concentrations of 10, 40, and 100 ng/mL were tested. Atomic force microscopy (AFM) imaging revealed a clear concentration-dependent increase in bead density on the sensor surface (Figure 5A), confirming successful sandwich complex formation and bead capture.

To identify the detection threshold, the magneto-immunoassay was performed using myoglobin antigen with eight different concentrations (50, 60, 65, 70, 75, 80, 85, and 90 ng/mL). The resulting dose–response curve is presented in Figure 5B. Under the optimal conditions (1 μm magnetic beads, 60 Oe external field), the ∆MR value at the clinical cut-off concentration of 75 ng/mL was 0.202%. This ∆MR75 value was therefore defined as the detection threshold. Samples producing ∆MR values greater than 0.202% were classified as positive for myoglobin. Specificity was assessed using BSA and troponin I (Figure 5C). The assay showed negligible cross-reactivity with these non-target proteins, while generating a strong positive signal for myoglobin. This result confirms that the assay is highly specific to myoglobin. To assess the impact of complex biological matrices, we conducted spiked experiments. When 90 ng/mL myoglobin was added to whole blood and plasma samples derived from healthy individuals, the GMR sensor response signals showed no significant difference compared to those measured in PBS buffer (*p* > 0.05). This indicates that the detection platform exhibits good resistance to matrix interference, with complex components in plasma and whole blood failing to significantly interfere with target detection. This preliminary validation confirms the method’s suitability for analyzing complex clinical samples.

## 4. Conclusions

This study presents an immunoassay platform that integrates magneto-immunosensing and microfluidic technologies. The platform consisted of a GMR sensor embedded in a microfluidic chip. The optimal parameters for the platform included 5% EDC + 5%NHS (activating agent), 1 mg/mL capture antibody, 1% BSA (blocking agent), 1 μm magnetic beads (detection carrier), 50 min of reaction time, and an external magnetic field of 60 Oe. Under these optimized parameters, the platform exhibited a ∆MR signal change of 0.202% at a myoglobin concentration of 75 ng/mL. Specificity tests demonstrated that the platform could distinguish myoglobin from non-target proteins such as bovine serum albumin and troponin I. The platform shows qualitative detection capability for myoglobin, supporting its potential use in early diagnosis applications.

This study has preliminarily validated the fundamental feasibility of an integrated GMR sensing platform, though certain limitations remain. To advance its practical application, subsequent work will focus on the following directions: (1) Achieving full automation of fluid drive, reaction control, and signal readout to reduce manual intervention, thereby enhancing the stability and throughput of the detection process; (2) Developing multi-marker simultaneous testing capabilities to detect key acute myocardial infarction biomarkers such as myoglobin and troponin, thereby addressing the insufficient specificity of single markers and enhancing diagnostic comprehensiveness and accuracy; (3) Establishing standardized quantitative methods and conducting systematic validation using real clinical samples to further enhance reliability in the early diagnosis of AMI. Through these optimizations, the technology holds promise as an effective technical support for rapid triage and precise intervention in acute myocardial infarction.

## Data Availability

The original contributions presented in this study are included in this article; further inquiries can be directed to the corresponding author.

## Supplementary Materials

The following supporting information can be downloaded at: https://www.mdpi.com/article/10.3390/bios16010008/s1, Figure S1: Physical image of the magnetically sensitive immunoassay platform; Figure S2: 3D schematic diagram of the microfluidic chip. References [32,33,34,35,36,37,38,39,40,41,42] are cited in the Supplementary Materials.
