# Supplementary material for: Integrated Microfluidic Giant Magnetoresistance (GMR) Biosensor Platform for Magnetoresistive Immunoassay of Myoglobin
Source: Biosensors (Basel). 2025 Dec 22;16(1):8. doi: 10.3390/bios16010008 (PMC12838720; doi:10.3390/bios16010008)
Supplement: Supplementary file 1 [file biosensors-16-00008-s001.zip › biosensors-4002769-supplementary.pdf]

biosensors

MDPI

Supplementary Materials

Integrated Microfluidic Giant Magnetoresistance (GMR) Biosensor Platform for Magnetoresistive Immunoassay of Myoglobin

Yikai Wang [1], Huaiyu Wang [2], Yunyun Zhang [2], Shuhui Cui [2], Fei Hu [2,*] and Bo'an Li [3,*]

1 Science and Technology Research Center of China Customs, Beijing 100026, China
2 State Key Laboratory for Manufacturing System Engineering, School of Instrument Science and Technology, Xi'an Jiaotong University, Xi'an 710049, China
3 Department of Clinical Laboratory, The Fifth Medical Center, Chinese People's Liberation Army (PLA) General Hospital, Beijing 100039, China
* Correspondence: hufei0701@mail.xjtu.edu.cn (F.H.); lba@263.net (B.L.)

Table of contents

Figure S1

Figure S2

Table S1

Signal Processing

To eliminate individual sensor differences and ensure result comparability, a standardized signal processing procedure was adopted in this study: first, the baseline resistance (R_0) of the sensor in buffer was collected, followed by the measurement of the stable resistance (R_signal) after the immunoreaction. The relative resistance change rate ($\Delta R/R_0$) was calculated and normalized against the intrinsic magnetoresistance (MR_0; the average value of sensors in this batch was approximately 1.8%), and the dimensionless parameter $\Delta MR/MR_0$ was finally used as the detection signal. This algorithm effectively eliminated inter-batch fluctuation interference by anchoring the response signal to the intrinsic performance baseline of each sensor.

Processing Methods for GMR Sensors

The sensor structure comprised three functional layers from bottom to top: a GMR sensing layer, a metal electrode layer, and a SiO_2 insulating protective layer. First, a spin valve film with an underlayer-pinned structure was fabricated on a SiO_2 substrate via magnetron sputtering, with a specific composition of Si/Ta(5)/FeNi(2)/IrMn(8)/CoFe(2)/Ru(0.8)/CoFe(2)/Cu(2.3)/CoFe(1.5)/FeNi(2)/Ta(3) (thickness unit: nm). Subsequently, the patterning process for the fine sensor lines was performed. Initially, a 200 μm-thick positive photoresist was spin-coated onto the substrate. After photolithographic exposure and development, a stepwise dry etching strategy was adopted to form GMR lines with a width of 200 μm. To prevent line breakage caused by thermal accumulation, an etching cycle of 3 min/3 min cooling was repeated three times to complete pattern transfer. During the photoresist removal process, to avoid line damage induced by ultrasonication, the photoresist was softened by soaking in acetone for 2–3 h, followed by gentle wiping for removal. Finally, the substrate was cleaned with deionized water and dried at 90 °C

for 2 h. The electrode layer was fabricated using the lift-off process: a 200 μm-thick photoresist was first spin-coated and patterned to expose the electrode and pin regions. After sputtering a Cr/Cu (100 nm) film, the photoresist was removed by soaking in acetone for 20 min, completing the electrode pattern definition. The SiO_2 protective layer (50 nm) was fabricated via the same lift-off process: after patterning, the insulating layer was sputtered, and finally baked at 90 °C for 2 h to ensure film stability.

Simulation Analysis of Microfluidics in Microfluidic Chips

Microfluidic chips require interfacing with a fluid drive module to control and propel fluid flow within the chip. The fluid drive module presented herein utilizes pneumatic actuation, with its pneumatic chamber connected to the microfluidic chip interface during operation. When the internal operating pressure of the chip is relatively high, the change in gas volume becomes significant and must be accounted for. Under such conditions, the influence of gas compressibility on the drive performance should be incorporated into the parameter calculations for the fluid drive module. Conversely, at very low operating pressures, the gas volume can be regarded as essentially constant, allowing the gas to be treated as an incompressible fluid in relevant calculations. Thus, to accurately evaluate the drive characteristics, it is essential to conduct a simulation analysis of the pressure distribution inside the microfluidic chip.

Prior to simulation, the microfluidic channel geometry within the chip must be determined. The equivalent diameter of the microchannels is calculated using the following formula:

$$D = \frac{2a^2}{(a + a)} \quad \text{(S1)}$$

In the equation: D—equivalent diameter of microchannel/mm;

a—cross-sectional edge length/mm.

Calculated value: D = 0.5 mm = 5 e^{-4} m.

The working fluid in the microfluidic chip is a PBS solution. With the fluid density assumed as $\rho = 1.03e^3$kg/m^3, fluid viscosity $\mu =$ e^{-3} Pa.s（at 20 °C）.

The fluid velocity at the inlet can be calculated by:

$$v = \frac{Q}{S} \quad \text{(S2)}$$

In the formula: Q—Flow rate /uL/s, set to 3 uL/s; S—Cross-sectional area at the inlet/mm^2.

$$S = \pi r^2 \quad \text{(S3)}$$

The calculated $S \approx 20mm^2$, then v is calculated as $v = 0.15$ mm/s $=$ 1.5e^{-4} m/s

Reynolds number Re calculation formula:

$$Re = \frac{\rho v d}{\mu} \quad \text{(S4)}$$

Where: Re—Reynolds number; ρ—fluid density, kg/m^3; v—fluid velocity, m/s; d—equivalent diameter, m; μ—dynamic viscosity, Pa·s.

The calculated $\mathrm{Re} = 7.725e^{-2}$, which is significantly less than the critical $Re_\alpha = 2300$. This confirms that the flow within the microfluidic chip is laminar. Indeed, the combination of low flow velocity and small channel dimensions typically results in laminar flow in microfluidic systems, as substantiated by the present calculation.

https://doi.org/ 10.3390/bios16010008

Based on the operational workflow of the microfluidic chip, a fluid dynamics simulation was conducted using COMSOL Multiphysics. The velocity value obtained from Equation (S3) was prescribed as the inlet boundary condition to compute the pressure and velocity fields throughout the chip.

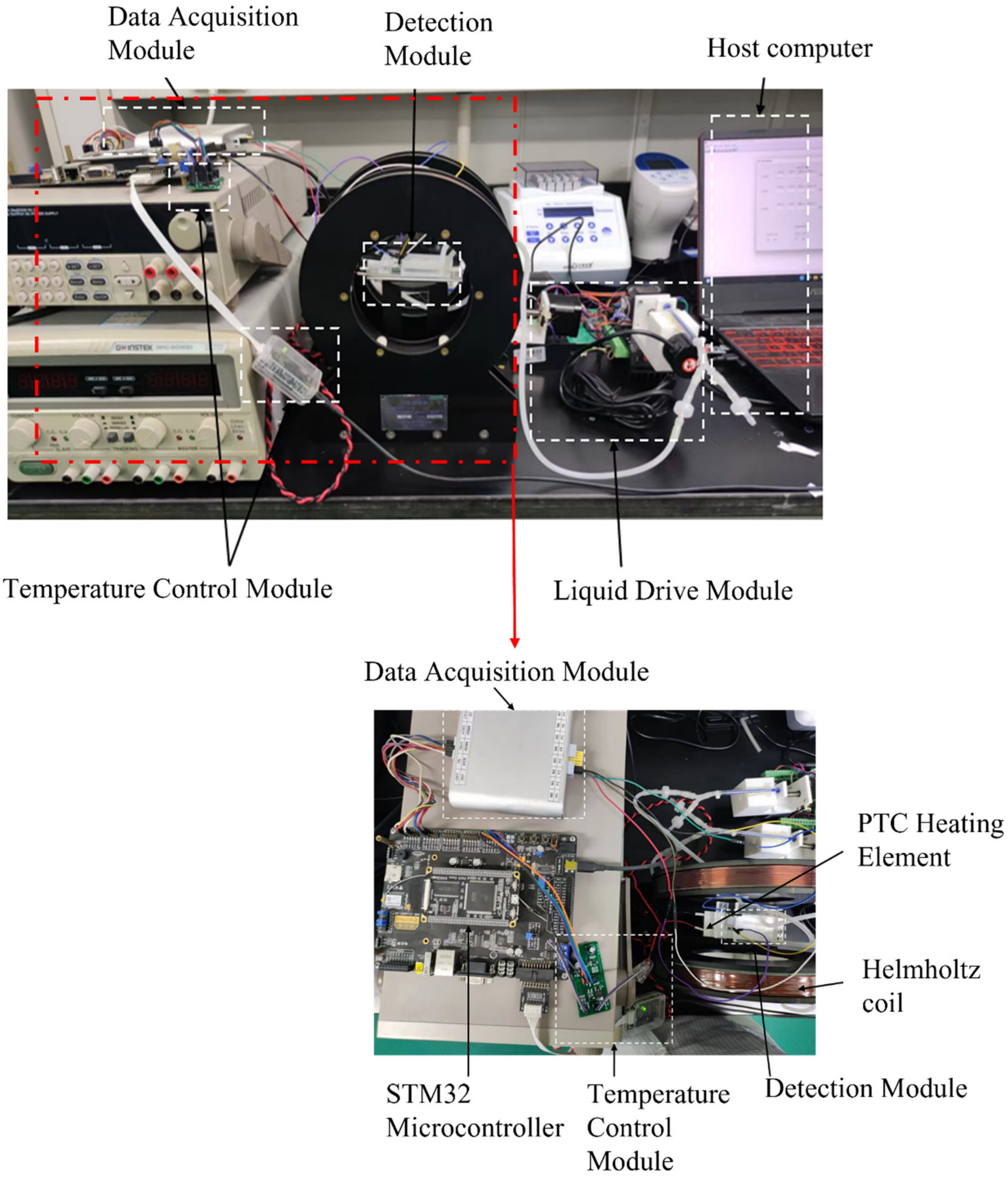

Figure S1. Physical image of the magnetically sensitive immunoassay platform. The system consists of a core detection module, a fluid drive module, a temperature control module, a signal acquisition module, a control module, a host computer, and an external magnetic field device.

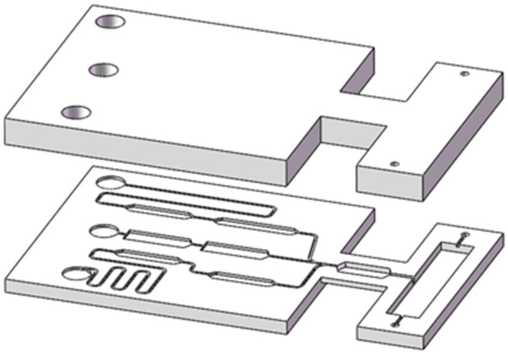

Figure S2. 3D schematic diagram of the microfluidic chip. The chip is formed by bonding an upper layer and a lower layer together.

Table S1. Comparison of Different Detection Methods.

Methods	Target	LOD	Sample types	Assay time	Reference
Electrochemical	Myoglobin	1 µg/ml	Human serum samples	< 1 h	[1]
Fluorescence	Myoglobin	<50 ng/ml	Human heart myoglobin	Not mentioned	[2]
Electrochemical	Myoglobin	34.6 ng/mL	human serum	Not mentioned	[3]
enzyme-linked immunoassay (ELISA)	Prostate specific antigen	0.008 µg/l	human serum	3.5 h	[4]
Chemiluminescence immunoassay (CLIA)	mIgG	0.1 µg/mL	mIgG protein	In 2 h	[5]
GMR	CEA	10 pg/mL	Human CEA antigen	Not mentioned	[6]
GMR	Influenza A virus	1.5×10^2 TCID50/mL	Swine nasal samples	50 min	[7]
TMR	E. coli O157:H7	100 CFU/mL	E. coli O157:H7 bacteria	In 5 h	[8]
GMR	CA125 II, HE4, and IL6	3.7 U/mL, 7.4 pg/mL, and 7.4 pg/mL	Protein	Several hours	[9]
Microcontact imprinted surface plasmon resonance sensor	Myoglobin	87.6 ng/mL	Human blood	Not mentioned	[10]
Portable Electrochemical Immunosensor	Human Epididymis Protein 4	3.5 pM	Human serum	>2 h	[11]
GMR	Myoglobin	<50 ng/ml	Myoglobin protein	~1 h	This study

References

1. Mandal, S.S.; Narayan, K.K.; Bhattacharyya, A.J. Employing denaturation for rapid electrochemical detection of myoglobin using TiO2 nanotubes. *J. Mater. Chem. B* **2013**, *1*, 3051–3056, https://doi.org/10.1039/c3tb20409j.
2. Darain, F.; Yager, P.; Gan, K.L.; Tjin, S.C. On-chip detection of myoglobin based on fluorescence. *Biosens. Bioelectron.* **2009**, *24*, 1744–1750, https://doi.org/10.1016/j.bios.2008.09.004.

3. Adeel, M.; Rahman, M.; Lee, J.-J. Label-free aptasensor for the detection of cardiac biomarker myoglobin based on gold nanoparticles decorated boron nitride nanosheets. Biosens. Bioelectron. 2019, 126, 143–150, https://doi.org/10.1016/j.bios.2018.10.060.
4. Matsumoto, K.; Konishi, N.; Hiasa, Y.; Kimura, E.; Takahashi, Y.; Shinohara, K.; Samori, T. A highly sensitive enzyme-linked immunoassay for serum free prostate specific antigen (f-PSA). *Clin. Chim. Acta* **1999**, *281*, 57–69, https://doi.org/10.1016/s0009-8981(98)00208-3.
5. Gupta, S.; Huda, S.; Kilpatrick, P.K.; Velev, O.D. Characterization and Optimization of Gold Nanoparticle-Based Silver-Enhanced Immunoassays. *Anal. Chem.* **2007**, *79*, 3810–3820, https://doi.org/10.1021/ac062341m.
6. Sun, X.-C.; Lei, C.; Guo, L.; Zhou, Y. Giant magneto-resistance based immunoassay for the tumor marker carcinoembryonic antigen. *Microchim. Acta* **2016**, *183*, 1107–1114, https://doi.org/10.1007/s00604-015-1686-2.
7. Krishna, V.D.; Wu, K.; Perez, A.M.; Wang, J.-P. Giant Magnetoresistance-based Biosensor for Detection of Influenza A Virus. *Front. Microbiol.* **2016**, 7, 400–400, https://doi.org/10.3389/fmicb.2016.00400.
8. Wu, Y.; Liu, Y.; Zhan, Q.; Liu, J.P.; Li, R.-W. Rapid detection of Escherichia coli O157:H7 using tunneling magnetoresistance biosensor. *Aip Adv.* **2017**, *7*, 056658. https://doi.org/10.1063/1.4977017
9. Klein, T.; Wang, W.; Yu, L.; Wu, K.; Boylan, K.L.M.; Vogel, R.I.; Skubitz, A.P.N.; Wang, J.-P. Development of a multiplexed giant magnetoresistive biosensor array prototype to quantify ovarian cancer biomarkers. *Biosens. Bioelectron.* **2019**, *126*, 301–307, doi:10.1016/j.bios.2018.10.046.
10. Osman, B.; Uzun, L.; Beşirli, N.; Denizli, A. Microcontact imprinted surface plasmon resonance sensor for myoglobin detection. *Mater. Sci. Eng. C* **2013**, *33*, 3609–3614, https://doi.org/10.1016/j.msec.2013.04.041.
11. Bianchi, V.; Mattarozzi, M.; Giannetto, M.; Boni, A.; De Munari, I.; Careri, M. A Self-Calibrating IoT Portable Electrochemical Immunosensor for Serum Human Epididymis Protein 4 as a Tumor Biomarker for Ovarian Cancer. *Sensors* **2020**, *20*, 2016, https://doi.org/10.3390/s20072016.
